# Contextual influence on confidence judgments in human reinforcement learning

**DOI:** 10.1371/journal.pcbi.1006973

**Published:** 2019-04-08

**Authors:** Maël Lebreton, Karin Bacily, Stefano Palminteri, Jan B. Engelmann

**Affiliations:** 1 CREED, Amsterdam School of Economics (ASE), Universiteit van Amsterdam, Amsterdam, the Netherlands; 2 Amsterdam Brain and Cognition (ABC), Universiteit van Amsterdam, Amsterdam, the Netherlands; 3 Neurology and Imaging of Cognition (LabNIC), Department of Basic Neurosciences, University of Geneva, Geneva, Switzerland; 4 Swiss Center for Affective Science (CISA), University of Geneva, Geneva, Switzerland; 5 Human Reinforcement Learning team, Université de Recherche Paris Sciences et Lettres, Paris, France; 6 Département d’Études Cognitives, École Normale Supérieure, Paris, France; 7 Laboratoire de Neurosciences Cognitives et Computationnelles, Institut National de la Santé et de la Recherche Médicale, Paris, France; 8 The Tinbergen Institute, Amsterdam, the Netherlands; UCL, UNITED KINGDOM

## Abstract

The ability to correctly estimate the probability of one’s choices being correct is fundamental to optimally re-evaluate previous choices or to arbitrate between different decision strategies. Experimental evidence nonetheless suggests that this metacognitive process—confidence judgment- is susceptible to numerous biases. Here, we investigate the effect of outcome valence (gains or losses) on confidence while participants learned stimulus-outcome associations by trial-and-error. In two experiments, participants were more confident in their choices when learning to seek gains compared to avoiding losses, despite equal difficulty and performance between those two contexts. Computational modelling revealed that this bias is driven by the context-value, a dynamically updated estimate of the average expected-value of choice options, necessary to explain equal performance in the gain and loss domain. The biasing effect of context-value on confidence, revealed here for the first time in a reinforcement-learning context, is therefore domain-general, with likely important functional consequences. We show that one such consequence emerges in volatile environments, where the (in)flexibility of individuals’ learning strategies differs when outcomes are framed as gains or losses. Despite apparent similar behavior- profound asymmetries might therefore exist between learning to avoid losses and learning to seek gains.

## Author summary

In order to arbitrate between different decision strategies, as well as to inform future choices, a decision maker needs to estimate the probability of her choices being correct as precisely as possible. Surprisingly, this metacognitive operation, known as confidence judgment, has not been systematically investigated in the context of simple instrumental-learning tasks. Here, we assessed how confident individuals are in their choices when learning stimulus-outcome associations by trial-and-errors to maximize gains or to minimize losses. In two experiments, we show that individuals are more confident in their choices when learning to seek gains compared to avoiding losses, despite equal difficulty and performance between those two contexts. To simultaneously account for this pattern of choices and confidence judgments, we propose that individuals learn context-values, which approximate the average expected-value of choice options. We finally show that, in volatile environments, the biasing effect of context-value on confidence induces difference in learning flexibility when outcomes are framed as gains or losses.

## Introduction

Simple reinforcement learning algorithms efficiently learn by trial-and-error to implement decision policies that maximize the occurrence of rewards and minimize the occurrence of punishments [1]. Such basic algorithms have been extensively used in experimental psychology, neuroscience and economics, and seem to parsimoniously account for a large amount of experimental data at the behavioral [2,3] and neuronal levels [4–6], as well as for learning abnormalities due to specific pharmacological manipulations [7,8] and neuro-psychiatric disorders [9]. Yet, ecological environments are inherently ever-changing, volatile and complex, such that organisms need to be able to flexibly adjust their learning strategies or to dynamically select among different learning strategies. These more sophisticated behaviors can be implemented by reinforcement-learning algorithms which compute different measures of environmental uncertainty [10–12] or strategy reliability [13–15].

To date, surprisingly little research has investigated if and how individuals engaged in learning by trial-and-error can actually compute such reliability estimates or related proxy variables. One way to experimentally assess such reliability estimates is via eliciting confidence judgments. Confidence is defined as a decision-maker’s estimation of her probability of being correct [16–18]. It results from a meta-cognitive operation [19], which according to recent studies could be performed automatically even when confidence judgments are not explicitly required [20]. In the context of predictive-inference tasks, individuals’ subjective confidence judgments have been shown to track the likelihood of decisions being correct in changing environments with remarkable accuracy [21,22]. Confidence could therefore be employed as a meta-cognitive variable that enables dynamic comparisons of different learning strategies and ultimately, decisions about whether to adjust learning strategies. Despite the recent surge of neural, computational and behavioral models of confidence estimation in decision-making and prediction tasks [17,23,24], how decision-makers estimate their confidence in their choices in reinforcement-learning contexts remains poorly investigated.

Crucially, although confidence judgments have been reported to accurately track decision-makers probability of being correct [18,22], they are also known to be subject to various biases. Notably, it appears that individuals are generally overconfident regarding their own performance [25], and that confidence judgments are modulated by numerous psychological factors including desirability biases [26], arousal [27], mood [28], and emotions [29] such as anxiety [30]. A recent study also revealed that monetary stakes can bias individuals’ confidence in their choice: irrespective of the choice correctness, the prospects of gains and losses bias confidence judgments upwards and downwards, respectively [31]. Given the potential importance of confidence in mediating learning strategies in changing environments, investigating confidence judgments and their biases in reinforcement-learning appears crucial.

Here, we simultaneously investigated the learning behavior and confidence estimations of individuals engaged in a reinforcement-learning task where the valence of the decision outcomes was systematically manipulated (gains versus losses) [8,32]. In this task, young adults have repeatedly been shown to perform equally well in gain-seeking and loss-avoidance learning contexts [32,33]. Yet, in line with the confidence bias induced by monetary stakes [31], we hypothesized that individuals would exhibit lower confidence in their choices while learning to avoid losses compared to seeking gains, despite similar performance and objectively equal difficulty between these two learning contexts. In addition, we anticipated that this bias would be generated by the learned *context-value*: this latent variable computed in some reinforcement-learning models–see e.g. [32,34]—approximates the overall expected value from available cues on a trial-by-trial basis, hence it could mimic the effects of the monetary stakes observed in [31]. Finally, conditional on those first hypotheses being confirmed, we hypothesized that the valence-induced confidence bias would modulate performance in volatile environments such as reversal tasks.

Our results, which confirm these hypotheses, first illustrate the generalizability of the confidence bias induced by the framing of incentives and outcomes as gains or losses. They also suggest that tracking confidence judgments in reinforcement-learning tasks can provide valuable insight into learning processes. Finally, they reveal that–despite apparent similar behavior- profound asymmetries might exist between learning to avoid losses and learning to seek gains [35], with likely important functional consequences.

## Results

### Experiment 1

**Fig 1:**
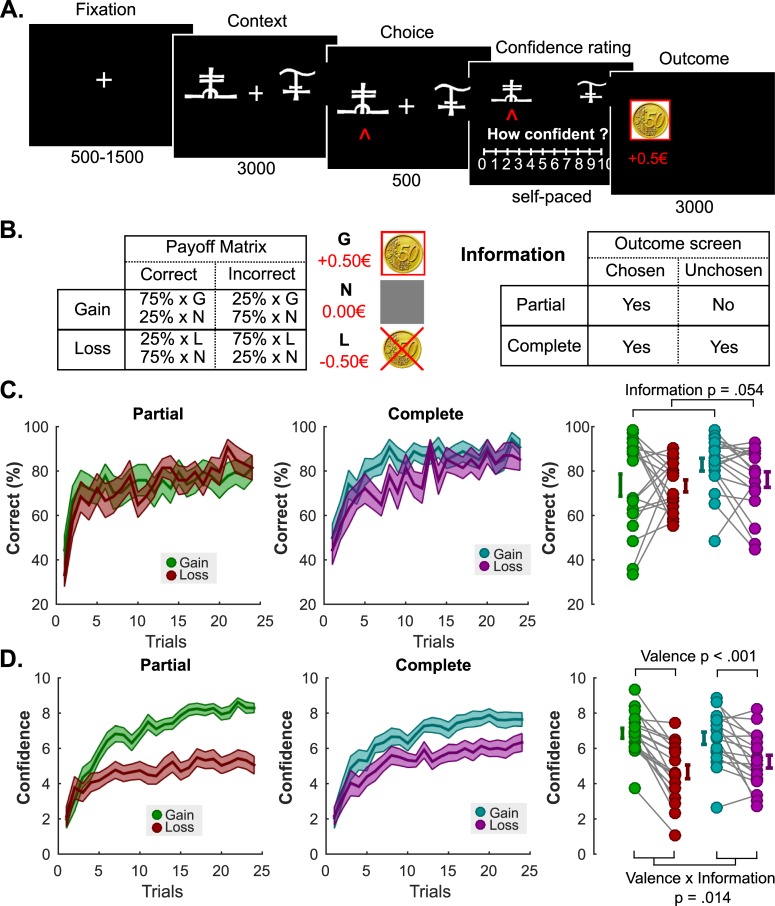
**Experiment 1 Task Schematic, Learning and Confidence Results (A) Behavioral task.** Successive screens displayed in one trial are shown from left to right with durations in ms. After a fixation cross, participants viewed a couple of abstract symbols displayed on both sides of a computer screen and had to choose between them. They were thereafter asked to report their confidence in their choice on a numerical scale (graded from 0 to 10). Finally, the outcome associated with the chosen symbol was revealed. (B) **Task design and contingencies.** (C) **Performance.** Trial by trial percentage of correct responses in the partial (left) and the complete (middle) information conditions. Filled colored areas represent mean ± sem; Right: Individual averaged performances in the different conditions. Connected dots represent individual data points in the within-subject design. The error bar displayed on the side of the scatter plots indicate the sample mean ± sem. (D) **Confidence.** Trial by trial confidence ratings in the partial (left) and the complete (middle) information conditions. Filled colored areas represent mean ± sem; Right: Individual averaged performances in the different conditions. Connected dots represent individual data points in the within-subject design. The error bar displayed on the side of the scatter plots indicate the sample mean ± sem.

We invited 18 participants to partake in our first experiment, and asked them to perform a probabilistic instrumental-learning task adapted from a previous study [32,33]. Participants repeatedly faced pairs of abstract symbols probabilistically associated with monetary outcomes. Symbol pairs were fixed, and associated with two levels of two outcome features, namely valence and information, in a 2×2 factorial design. Therefore, pairs of symbols could be associated with either gains or losses, and with partial or complete feedback (**Methods** and **Fig 1A and 1B**). Participants could maximize their payoffs by learning to choose the most advantageous symbol of each pair, i.e., the highest expected gain or the lowest expected loss. At each trial, after their choice but before receiving feedback, participants were also asked to report their confidence in their choice on a Likert scale from 0 to 10. Replicating previous findings [32,33], we found that participants correctly learned by trial-and-error to choose the best outcomes, (average correct choice rate 76.50 ± 2.38, t-test vs chance t_17_ = 11.16; P = 3.04×10^−9^), and that learning performance was marginally affected by the information factor, but unaffected by the outcome valence (ANOVA; main effect of information F_1,17_ = 4.28; P = 0.05; main effect of valence F_1,17_ = 1.04; P = 0.32; interaction F_1,17_ = 1.06; P = 0.32; **Fig 1C**). In other words, participants learned equally well to seek gains and to avoid losses. However, and in line with our hypothesis, the confidence ratings showed a very dissimilar pattern, as they were strongly influenced by the valence of outcomes (ANOVA; main effect of information F_1,17_ = 2.00; P = 0.17; main effect of valence F_1,17_ = 33.11; P = 2,33×10^−11^; interaction F_1,17_ = 7.58; P = 0.01; **Fig 1D**). Similar to the valence bias reported in perceptual decision-making tasks [31], these effects were driven by the fact that participants were more confident in the gain than in the loss condition when receiving partial feedback (6.86 ± 0.28 vs 4.66 ± 0.39; t-test t_17_ = 7.20; P = 1.50×10^−6^), and that this difference was still very significant although smaller in the complete feedback condition (6.58 ± 0.35 vs 5.24 ± 0.37; t-test t_17_ = 3.52; P = 2.65×10^−3^).

### Experiment 2

**Fig 2:**
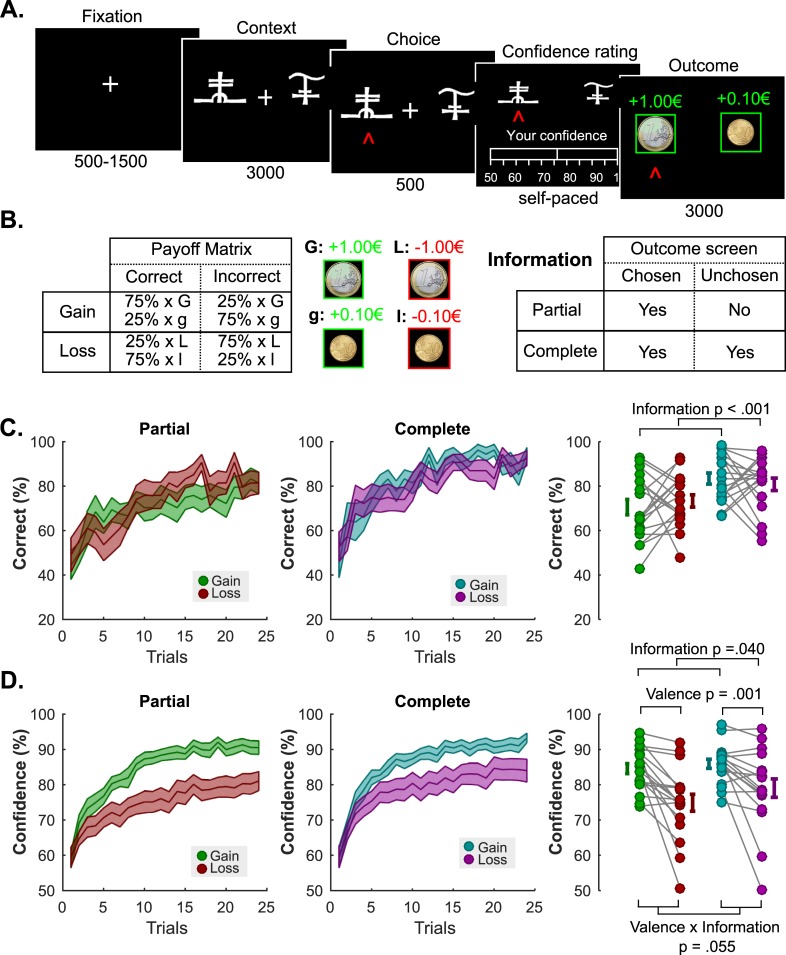
**Experiment 2 Task Schematic, Learning and Confidence Results (A) Behavioral task.** Successive screens displayed in one trial are shown from left to right with durations in ms. After a fixation cross, participants viewed a couple of abstract symbols displayed on both sides of a computer screen, and had to choose between them. They were thereafter asked to report their confidence in their choice on a numerical scale (graded from 50 to 100%). Finally, the outcome associated with the chosen symbol was revealed. (B) **Task design and contingencies.** (C) **Performance.** Trial by trial percentage of correct responses in the partial (left) and the complete (middle) information conditions. Filled colored areas represent mean ± sem; Right: Individual averaged performances in the different conditions. Connected dots represent individual data points in the within-subject design. The error bar displayed on the side of the scatter plots indicate the sample mean ± sem. (D) **Confidence.** Trial by trial confidence ratings in the partial (left) and the complete (middle) information conditions. Filled colored areas represent mean ± sem; Right: Individual averaged performances in the different conditions. Connected dots represent individual data points in the within-subject design. The error bar displayed on the side of the scatter plots indicate the sample mean ± sem.

**Fig 3:**
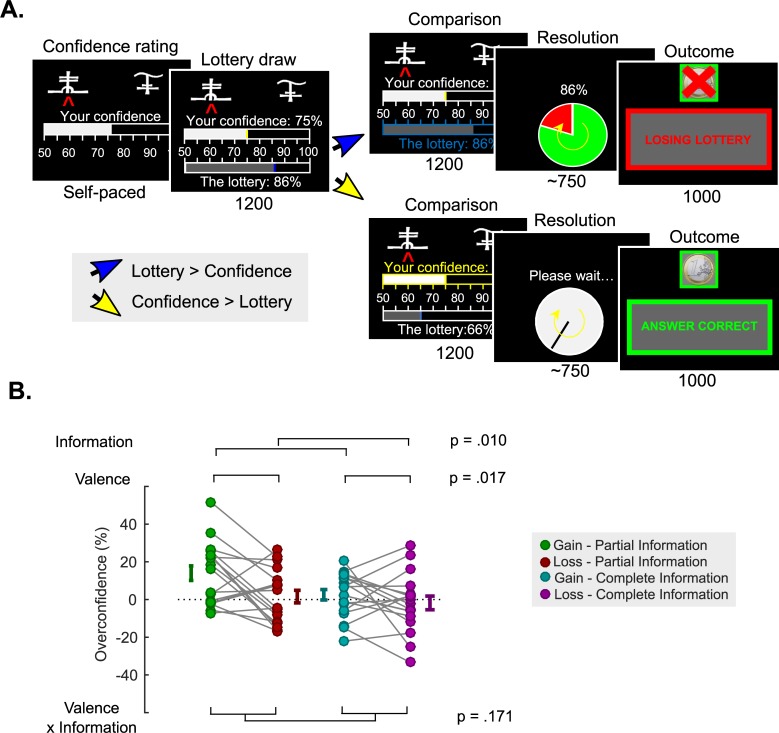
**Incentive mechanism and overconfidence (A) Incentive mechanism.** In Experiment 2, for the payout-relevant trials a lottery L is randomly drawn in the 50–100% interval and compared to the confidence rating C. If L > C, the lottery is implemented. A wheel of fortune, with a L% chance of losing is displayed, and played out. Then, feedback informed participants whether the lottery resulted in a win or a loss. If C > L, a clock is displayed together with the message “Please wait”, followed by feedback which depended on the correctness of the initial choice. With this mechanism, participant can maximize their earning by reporting their confidence accurately and truthfully. (B) **Overconfidence**. Individual averaged calibration, as a function of Experiment 2 experimental conditions (with a similar color code as in **Figs 1 and 2**). Connected dots represent individual data points in the within-subject design. The error bar displayed on the side of the scatter plots indicate the sample mean ± sem.

While the results of the first experiment are strongly suggestive of an effect of outcome valence on confidence in reinforcement learning, they cannot *formally* characterize a bias, as the notion of cognitive bias depends on the optimal reward-maximizing strategy [36]. In other terms: does this bias persist in situations where a truthful and accurate confidence report is associated with payoff maximization? We addressed this limitation of experiment 1 by directly incentivizing reports of confidence accuracy in our follow-up experiment. In this new experiment, confidence was formally defined as an estimation of the probability of being correct, and participants could maximize their chance to gain an additional monetary bonus (3×5 euros) by reporting their confidence as accurately and truthfully as possible on a rating scale ranging from 50% to 100% (**Fig 2A**). Specifically, confidence judgments were incentivized with a Matching Probability (MP) mechanism, a well-validated method from behavioral economics adapted from the Becker-DeGroot-Marschak auction [37,38]. Briefly, the MP mechanism considers participants’ confidence reports as bets on the correctness of their answers, and implements comparisons between these bets and random lotteries (**Fig 3A**). Under utility maximization assumptions, this guarantees that participants maximize their earnings by reporting their most precise and truthful confidence estimation [39,40]. This mechanism and the dominant strategy were explained to the 18 new participants before the experiment (**Methods**). In addition, because the neutral and non-informative outcome was more frequently experienced in the punishment partial than in the reward partial context in experiment 1, we replaced the neutral 0€ with a 10c gain or loss (see **Methods** and **Fig 2B**).

Replicating the results from the first experiment, we found that learning performance was affected by the information factor, but unaffected by the outcome valence (ANOVA; main effect of information F_1,17_ = 18.64; P = 4.67×10^−4^; main effect of valence F_1,17_ = 1.33×10^−3^; P = 0.97; interaction F_1,17_ = 0.77; P = 0.39; **Fig 2C**). Yet, the confidence ratings were again strongly influenced by the valence of outcomes (ANOVA; main effect of information F_1,17_ = 4.92; P = 0.04; main effect of valence F_1,17_ = 15.43; P = 1.08×10^−3^; interaction F_1,17_ = 4.25; P = 0.05; **Fig 2D**). Similar to Experiment 1, these effects were driven by the fact that participants were more confident in the gain than in the loss conditions (85.25 ± 1.23 vs 76.96 ± 2.38 (in %); t-test t_17_ = 3.93; P = 1.08×10^−3^).

Importantly, the changes in the experimental design also allowed us to estimate the bias in confidence judgments (sometimes called calibration, or “overconfidence”), by contrasting individuals’ average reported confidence (i.e. estimated probability of being correct) with their actual average probability of being correct. A positive bias therefore indicates that participants are overconfident reporting a higher probability of being correct than their objective average performance. Conversely, a negative bias indicates reporting a lower probability of being correct than the true average (“underconfidence”). These analyses revealed that participants are, in general marginally overconfident (4.07 ± 2.37 (%); t-test vs 0: t_17_ = 1.72; P = 0.10). This overconfidence, which was maximal in the gain-partial information condition (14.00 ± 3.86 (%)), was nonetheless mitigated by complete information (gain-complete: 2.53 ± 2.77 (%); t-test vs gain-partial: t_17_ = 2.72; P = 0.01) and losses (loss-partial: 1.56 ± 3.35 (%); t-test vs gain-partial: t_17_ = 2.76; P = 0.01). These effects of outcome valence and counterfactual feedback information on overconfidence appeared to be simply additive (ANOVA; main effect of information F_1,17_ = 8.40; P = 0.01; main effect of valence F_1,17_ = 7.03; P = 0.02; interaction F_1,17_ = 2.05; P = 0.17; **Fig 3B**).

### Context-dependent learning

While the results from our two first experiments provide convincing support for our hypotheses at the aggregate level (i.e. averaged choice rate and confidence ratings), we aimed at providing a finer description of the dynamical processes at stake, and therefore turned to computational modelling. Standard reinforcement-learning algorithms [1,3] typically give a satisfactory account of learning dynamics in stable contingency tasks as ours, but recent studies [32–34] have demonstrated that human learning is highly context (or reference)-dependent. The specific context-dependent reinforcement-learning algorithm proposed to account for learning and post-learning choices in the present task explicitly computes a context-value, which approximates the average expected value from a specific context [32]. We therefore hypothesized that this latent variable would capture the effects of monetary stakes observed in our previous study [31] and bias confidence. While this hypothesis about confidence will be explicitly tested in the in the next section, we first aim to demonstrate in the present section that context-dependent learning is necessary to explain choices.

Context dependency, by allowing neutral or moderately negative outcomes to be reframed as relative gains, provides an effective and parsimonious solution to the punishment-avoidance paradox. Briefly, this paradox stems from the notion that once a punishment is successfully avoided, the instrumental response is no longer reinforced. Reward learning (in which the extrinsic reinforcements are frequent, because they are sought) should therefore, theoretically, be more efficient than punishment learning (in which the extrinsic reinforcements are infrequent, because they are avoided). Yet, human subjects have repeatedly been shown to learn equally well in both domains, which paradoxically contradicts this prediction [41]. Reframing successful punishment-avoidance as a relative gain in context-dependent learning models solves this punishment-avoidance paradox.

**Fig 4:**
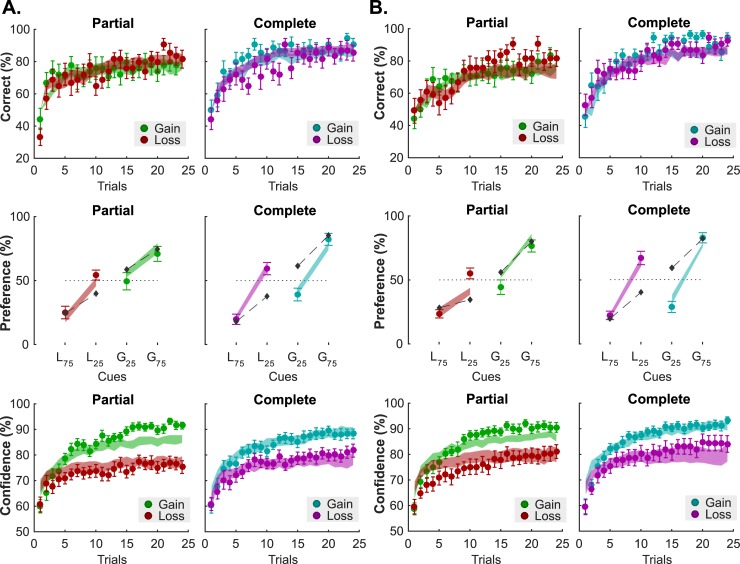
Modelling results: Fits. Behavioral results and model fits in Experiments 1(**A**) and 2 (**B**). Top: Learning performance (i.e. percent correct). Middle: Choice rate in the transfer test. Symbols are ranked by expected value (L_75_: symbol associated with 75% probability of losing 1€; L_25_: symbol associated with 25% probability of losing 1€; G_25_: symbol associated with 25% probability of winning 1€; G_75_: symbol associated with 75% probability of winning 1€;) Bottom: Confidence ratings. In all panels, colored dots and error bars represent the actual data (mean ± sem), and filled areas represent the model fits (mean ± sem). Model fits were obtained with the RELATIVE reinforcement-learning model for the learning performance (top) and the choice rate in the transfer test (middle), and with the FULL glme for the confidence ratings (bottom). Dark grey diamonds in the Preference panels (middle) indicate the fit from the ABSOLUTE model.

Typically, implementing context dependency during learning generates “irrational” preferences in a transfer task performed after learning: participants express higher preference for mildly unfavorable items to objectively better items, because the former were initially paired with unfavorable items and hence acquired a higher “relative” subjective value [32–34]. As in these previous studies, the participants from our two experiments also performed the transfer task after the learning task (see **Methods**). The typical behavioral signature of context-dependent learning is a preference reversal in the complete information contexts, where symbols associated with small losses (L_25_) are preferred to symbols associated with small gains (G_25_), despite having objectively lower expected value [32–34]. This pattern was present in both of our experiments (% choices; experiment 1: L_25_: 59.52 ± 4.88, G_25_: 38.89 ± 5.04; t-test t_17_ = 2.46; P = 0.02; experiment 2: L_25_: 67.26 ± 5.35, G_25_: 28.37 ± 4.46; t-test t_17_ = 5.27; P = 6.24×10^−5^, see **Fig 4A and 4B**, middle panels).

To confirm these observations, we adopted a model-fitting and model-comparison approach, where a standard learning model (ABSOLUTE) was compared to a context-dependent learning model (RELATIVE) in its ability to account for the participants’ choices (**Methods**). Replicating previous findings [32,33], the context-dependent model provided the best and most parsimonious account of the data collected in our 2 experiments (**Table 1**), and a satisfactory account of choice patterns in both the learning (average likelihood per trial in experiment 1: 0.72 ± 0.03; in experiment 2: 0.72 ± 0.02; see **Fig 4A and 4B**, top panels) and transfer tasks (average likelihood per trial; experiment 1: 0.71 ± 0.02; experiment 1: 0.70 ± 0.02; see **Fig 4A and 4B**, middle panels). Please also note that the model estimated free-parameters (**Table 2**) are very similar to what was reported in the previous studies [32,33].

**Table 1:** Reinforcement-learning. **Model comparison.** AIC, Akaike Information Criterion (computed with nLL_max_); BIC, Bayesian Information Criterion (computed with nLL_max_); DF, degrees of freedom; nLL_max_, negative log likelihood; nLPP_max_, negative log of posterior probability; EF, expected frequency of the model given the data; XP, exceedance probability (computed using the Laplace approximation of the model evidence ME). The table summarizes for each model its fitting performances.

**Exp. 1**	**Model**	**DF**	-2*nLL_**max**_	2*AIC	**BIC**	-2*nLPP_**max**_	**EF**	**XP**
	ABSOLUTE	3	385±20	392±20	404±20	391±20	0.28	0.02
	RELATIVE	4	345±24	353±24	369±24	354±24	0.72	0.98
**Exp. 2**	**Model**	**DF**	-2*nLL_**max**_	2*AIC	**BIC**	-2*nLPP_**max**_	**EF**	**XP**
	ABSOLUTE	3	411±15	417±15	429±15	416±15	0.05	0.0
	RELATIVE	4	355±16	363±16	379±16	362±16	0.95	1.0

**Table 2:** Reinforcement-learning. **Free parameters.** ABSOLUTE, absolute value learning model; RELATIVE, relative value learning model (best-fitting model); LL optimization, parameters obtained when minimizing the negative log likelihood; LPP optimization, parameters obtained when minimizing the negative log of the posterior probability. The table summarizes for each model the likelihood maximizing (best) parameters averaged across subjects. Data are expressed as mean±s.e.m. The values retrieved from the LPP optimization procedure are those used to generate the variable used in the confidence glme models.

		**LL Optimization**		**LPP Optimization**	
**Exp. 1**	**Free Parameter**	ABSOLUTE	RELATIVE	ABSOLUTE	RELATIVE
	Inverse temperature (*β*)	6.29±0.63	54.04±38.8	6.07±0.61	12.65±1.47
	Factual learning rate (*α*_*c*_)	0.37±0.05	0.23±0.04	0.36±0.04	0.24±0.04
	Counterfactual learning rate (*α*_*u*_)	0.13±0.03	0.07±0.02	0.15±0.03	0.09±0.02
	Context learning rate (*α*_*V*_)	-	0.46±0.10	-	0.46±0.10
		**LL Optimization**		**LPP Optimization**	
**Exp. 2**	**Free Parameter**	ABSOLUTE	RELATIVE	ABSOLUTE	RELATIVE
	Inverse temperature (*β*)	102.00±99.49	83.05±73.15	2.65±0.29	6.86±0.81
	Factual learning rate (*α*_*c*_)	0.49±0.07	0.26±0.04	0.49±0.07	0.24±0.04
	Counterfactual learning rate (*α*_*u*_)	0.24±0.08	0.12±0.04	0.24±0.08	0.13±0.03
	Context learning rate (*α*_*V*_)	-	0.41±0.09	-	0.40±0.09

### A descriptive model of confidence formation

We next used latent variables from this computational model, along with other variables known to inform confidence judgments, to inform a descriptive model of confidence formation. We propose confidence to be under the influence of three main variables, entered as explanatory variables in linear mixed-effect regressions (FULL model–see **Methods. Confidence Model**). The first explanatory variable is choice difficulty, a feature captured in value-based choices by the absolute difference between the expected value of the two choice options [42,43], and indexed by the absolute difference between the option Q-values calculated by the RELATIVE model. The second explanatory variable is the confidence expressed at the preceding trial. Confidence judgments indeed exhibit a strong auto-correlation, even when they relate to decisions made in different tasks [44]. Note that in our task, where the stimuli are presented in an interleaved design, this last term captures the features of confidence which are transversal to different contexts such as aspecific drifts due to attention fluctuation and/or fatigue. The third and final explanatory variable is V(s), the approximation of the average expected-value of a pair of stimuli (i.e., the context value from the RELATIVE model) [32]. The context value, initialized at zero, gradually becomes positive in the reward-seeking conditions and negative in the punishment-avoidance conditions. This variable is central to our hypothesis that the decision frame (gain vs. loss) influences individuals’ estimated confidence about being correct [31]. Crucially, in the FULL model, all included explanatory variables were significant predictors of confidence ratings in both experiments (see **Table 3**). As a quality check, we also verified that the confidence ratings estimated under the FULL model satisfactorily capture the evolution of observed confidence ratings across the course of our experiments (**Fig 4A and 4B**, bottom panels).

**Table 3:** Modelling confidence ratings. Estimated fixed-effect coefficients from generalized linear mixed-effect models.

		**GLME**		
**Experiment 1**	**Fixed-Effect**	**REDUCED 1**	**REDUCED 2**	**FULL**
	Intercept (*β*_0_)	0.52±0.04 t_5079_ = 14.46; P = 1.90×10^−46^	0.72±0.02 t_5124_ = 39.00; P = 1.61×10^−291^	0.53±0.04 t_5078_ = 14.55; P = 4.92×10^−47^
	Choice difficulty (*β*_Δ*Q*_)	0.33±0.06 t_5079_ = 5.77; P = 8.43×10^−9^	0.47±0.07 t_5124_ = 6.51; P = 8.18×10^−11^-	0.30±0.05 t_5078_ = 5.96; P = 2.73×10^−9^
	Preceding confidence (*β*_*c*1_)	0.28±0.04 t_5079_ = 7.60; P = 3.62×10^−14^	-	0.28±0.03 t_5078_ = 7.39; P = 1.67×10^−13^
	Context value (*β*_*V*_)	-	0.45±0.14 t_5124_ = 3.16; P = 1.58×10^−3^	0.47±0.14 t_5078_ = 3.21; P = 1.35×10^−3^
		**GLME**		
**Experiment 2**	**Fixed-Effect**	**REDUCED 1**	**REDUCED 2**	**FULL**
	Intercept (*β*_0_)	0.53±0.03 t_5145_ = 17.57; P = 3.77×10^−67^	0.75±0.02 t_5145_ = 44.91; P = 0	0.53±0.03 t_5144_ = 17.12; P = 5.94×10^−64^
	Choice difficulty (*β*_Δ*Q*_)	0.18±0.02 t_5145_ = 6.33; P = 2.63×10^−10^	0.25±0.04 t_5145_ = 6.51; P = 8.26×10^−11^	0.17±0.03 t_5144_ = 5.90; P = 3.85×10^−9^
	Preceding confidence (*β*_*c*1_)	0.29±0.04 t_5145_ = 7.01; P = 2.75×10^−12^	-	0.30±0.04 t_5144_ = 7.48; P = 8.54×10^−14^
	Context value (*β*_*V*_)	-	0.17±0.7 t_5145_ = 2.52; P = 1.18×10^−2^	0.16±0.06 t_5144_ = 2.51; P = 1.19×10^−2^

Note that the number of degrees-of-freedom differs between REDUCED GLME 1 and 2 in Experiment 1, because some participants failed to answer within the allocated time, causing missed observations. This has a lower impact on the number of usable observations in the REDUCED GLME 2 because this model does not make use of “preceding confidence” (which are missing observations–in addition to the missed trials- in the REDUCED GLME 2 and FULL FLME).

On the contrary, when attempting to predict the trial-by-trial correct answers (i.e. performance) rather than confidence judgments with the same explanatory variables, the choice difficulty and the confidence expressed at the preceding trial were significant predictors in the two experiments, while the context value was not (**Table 4**). This again captures the idea that context value might bias confidence judgments above and beyond the variation in performance. Finally, because decision reaction times are known to be (negatively) correlated with subsequent confidence judgments—the more confident individuals are in their choices, the faster their decisions [20,42,45]-, we anticipated and verified that the same explanatory variables which are significant predictors of confidence also predict reaction times (although with opposite signs–see **Table 4**).

**Table 4:** Modelling performance and reaction times. Estimated fixed-effect coefficients from generalized linear mixed-effect models (performance: logistic regression; reaction times: linear regression).

		**GLME**	
**Experiment 1**	**Fixed-Effect**	**PERFORMANCE**	**RT**
	Intercept (*β*_0_)	-0.84±0.20 t_5078_ = -4.15; P = 3.40×10^−5^	1.90±0.09 t_5078_ = 20.12; P = 1.12×10^−86^
	Choice difficulty (*β*_Δ*Q*_)	9.90±1.67 t_5078_ = 5.92; P = 3.32×10^−9^	-0.65±0.20 t_5078_ = -3.15; P = 1.63×10^−3^
	Preceding confidence (*β*_*c*1_)	1.28±0.36 t_5078_ = 3.60; P = 3.19×10^−4^	-0.24±0.14 t_5078_ = -1.78; P = 0.08
	Context value (*β*_*V*_)	1.19±0.54 t_5078_ = 2.19; P = 0.03	-0.37±0.11 t_5078_ = -3.48; P = 5.04×10^−4^
		**GLME**	
**Experiment 2**	**Fixed-Effect**	**PERFORMANCE**	**RT**
	Intercept (*β*_0_)	-0.71±0.22 t_5144_ = -3.20; P = 1.37×10^−3^	1.68±0.09 t_5144_ = 17.93; P = 9.09×10^−70^
	Choice difficulty (*β*_Δ*Q*_)	5.29±0.76 t_5144_ = 6.94; P = 4.49×10^−12^	-0.41±0.09 t_5144_ = -4.50; P = 6.81×10^−6^
	Preceding confidence (*β*_*c*1_)	1.21±0.33 t_5144_ = 3.66; P = 2.57×10^−4^	-0.54±0.10 t_5144_ = -5.31; P = 1.08×10^−7^
	Context value (*β*_*V*_)	0.30±0.28 t_5144_ = 1.05; P = 0.29	-0.17±0.05 t_5144_ = -3.68; P = 2.35×10^−4^

#### Context values explain the confidence bias

In this last section, we aimed at demonstrating that the context values are necessary and sufficient to explain the difference in confidence observed between the reward seeking and the loss avoidance conditions. We therefore built a REDUCED model 1, which was similar to the FULL model, but lacked the context value (see **Table 3**). First, because the REDUCED model 1 is nested in the FULL model, a likelihood ratio test statistically assesses the probability of observing the estimated fitting difference under the null hypothesis that the FULL model is not better than the REDUCED model 1. In both experiments, this null hypothesis was rejected (both P<0.001), indicating that the FULL model provides a better explanation of the observed data. Hence confidence is critically modulated by the context value.

**Fig 5:**
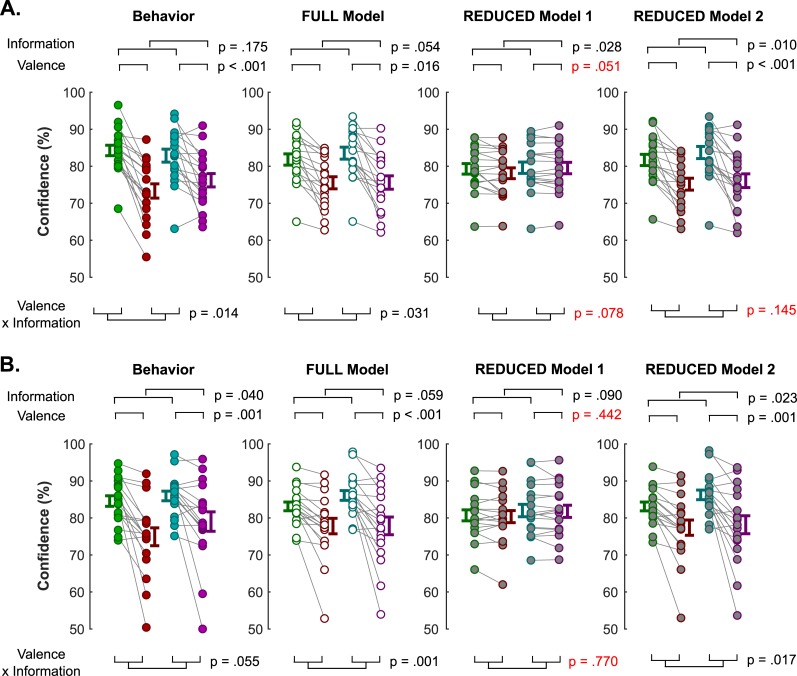
Modelling results: Lesioning approach. Three nested models are compared in their ability to reproduce the pattern of interest observed in averaged confidence ratings, in experiment 1 (**A**) and experiment 2 (**B**). In the FULL model, confidence is modelled as a function of three factors: the absolute difference between options values, the confidence observed in the previous trial, and the context value. In the REDUCED model 1, confidence is modelled as a function of only two factors: the absolute difference between options values and the confidence observed in the previous trial. Hence, the REDUCED model 1 omits the context-value as a predictor of confidence. In the REDUCED model 2, confidence is modelled as a function of only two factors: the absolute difference between options values and the context-value. Hence, the REDUCED model 2 omits the confidence observed in the previous trial as a predictor of confidence. Left: pattern of confidence ratings observed in the behavioral data. Middle-left: pattern of confidence ratings estimated from the FULL model. Middle-right: pattern of confidence ratings estimated from the REDUCED model 1. Right: pattern of confidence ratings estimated from the REDUCED model 2. In red are reported statistics from a repeated-measure ANOVA where the alternative model fails to reproduce important statistical properties of confidence observed in the data. Connected dots represent individual data points in the within-subject design. The error bar displayed on the side of the scatter plots indicate the sample mean ± sem.

Then, to demonstrate that the biasing effect of outcome valence on confidence is operated through the context value, we show that the REDUCED model 1 (see **Methods** for a detailed model description), which only lacks the context value as an explanatory variable, cannot reproduce the critical pattern of valence-induced confidence biases observed in our data, while the FULL model can (**Fig 5**) [46].

We performed similar analyses with a REDUCED model 2 (see **Methods** for a detailed model description), which only lacked the dependence on preceding confidence ratings. While this model could reproduce the valence-induced bias in confidence (**Fig 5**), likelihood ratio tests again rejected the hypothesis that the FULL model is not better than the REDUCED model 2 in both experiments (both P<0.001). Overall, those analyses demonstrate that both context-value and preceding confidence are necessary variables to explain confidence, context value being the crucial factor necessary to explain the valence-induced bias.

**Fig 6:**
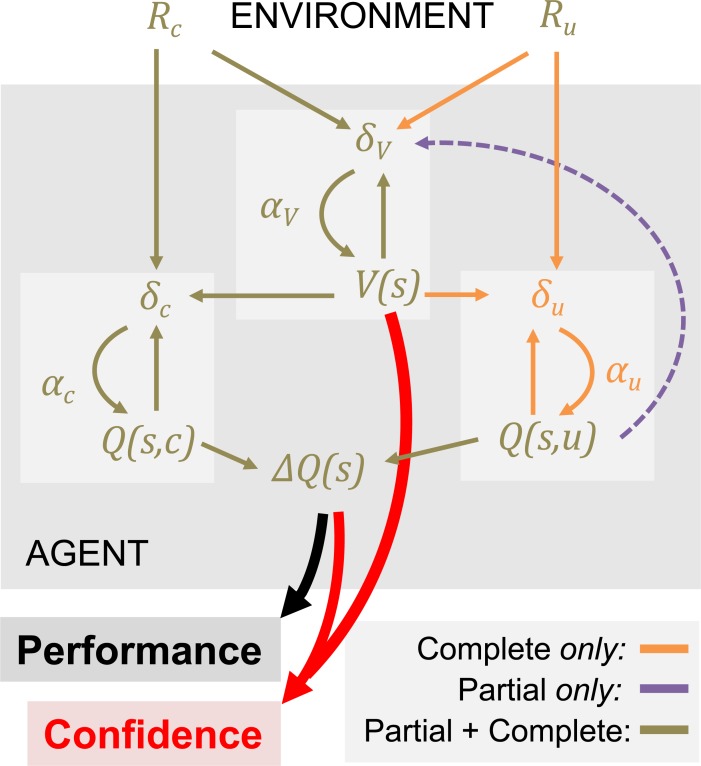
Summary of the modelling results. The schematic illustrates the computational architecture that best accounts for the choice and confidence data. In each context (or state) ‘s’, the agent tracks option values (Q(s,:)), which are used to decide amongst alternative courses of action, together with the value of the context (V(s)), which quantify the average expected value of the decision context. In all contexts, the agent receives an outcome associated with the chosen option (R_c_), which is used to update the chosen option value (Q(s,c)) via a prediction error (δ_c_) weighted by a learning rate (α_c_). In the complete feedback condition, the agent also receives information about the outcome of the unselected option (R_u_), which is used to update the unselected option value (Q(s,u)) via a prediction error (δ_u_) weighted by a learning rate (α_u_). The available feedback information (R_c_ and R_u_, in the complete feedback contexts and Q(s,u) in the partial feedback contexts) is also used to update the value of the context (V(s)), via a prediction error (δ_V_) weighted by a specific learning rate (α_V_). Option and context values jointly contribute to the generation of confidence judgments.

Overall, these results provide additional evidence for the importance of context value as an important latent variable in learning, not only explaining irrational choices in transfer tests, but also confidence biases observed during learning (**Fig 6**).

#### Assessing the specific role of context values in biasing confidence

So far, our investigations show that including context values (V(s)) as a predictor of confidence is necessary and sufficient to reproduce the bias in confidence induced by the decision frame (gain vs. loss). However, it remains unclear how specific and robust the contribution of context-values in generating this bias is, notably when other valence-sensitive model-free and model-based variable are accounted for. To address this question, we run two additional linear models: one including the sum of the two q-values (∑Q), which also tracks aspects of the valence of the context; the second including RTs, which were also predicted by both ΔQ and V(s) (see previous paragraph). In both experiments and for both linear models, the residual effect of V(s) on trial-by-trial confidence judgments remained positive and (marginally) significant (see **Table 5**), thus indicating a specific role of our model-driven estimate of V(s) above and beyond other related variables.

**Table 5:** Assessing the specific role of context values on confidence. Estimated fixed-effect coefficients from generalized linear mixed-effect models.

		**GLME**	
**GLME 1**	**Fixed-Effect**	**Experiment 1**	**Experiment 2**
	Intercept (*β*_0_)	0.58±0.05 t_5077_ = 18.06; P = 1.01×10^−70^	0.68±0.03 t_5143_ = 21.74; P = 2.48×10^−100^
	Choice difficulty (*β*_Δ*Q*_)	0.27±0.05 t_5077_ = 5.55; P = 2.97×10^−8^	0.13±0.03 t_5143_ = 4.97; P = 6.76×10^−7^
	Preceding confidence (*β*_*c*1_)	0.26±0.03 t_5077_ = 7.56; P = 4.79×10^−14^	0.24±0.04 t_5143_ = 6.93; P = 4.69×10^−12^
	Context value (*β*_*V*_)	0.43±0.14 t_5077_ = 3.14; P = 1.68×10^−3^	0.15±0.06 t_5143_ = 2.36; P = 1.81×10^−2^
	Reaction times (*β*_*RT*_)	-0.03±0.01 t_5077_ = -2.53; P = 1.15×10^−2^	-0.09±0.01 t_5143_ = -9.95; P = 4.04×10^−24^
		**GLME**	
**GLME 2**	**Fixed-Effect**	**Experiment 1**	**Experiment 2**
	Intercept (*β*_0_)	0.53±0.04 t_5077_ = 14.99; P = 9.36×10^−50^	0.53±0.03 t_5143_ = 16.83; P = 6.45×10^−62^
	Choice difficulty (*β*_Δ*Q*_)	0.24±0.05 t_5077_ = 4.59; P = 4.53×10^−6^	0.14±0.03 t_5143_ = 4.79; P = 1.75×10^−6^
	Preceding confidence (*β*_*c*1_)	0.28±0.04 t_5077_ = 7.50; P = 7.30×10^−14^	0.30±0.04 t_5143_ = 7.70; P = 1.60×10^−14^
	Context value (*β*_*V*_)	0.10±0.05 t_5077_ = 1.94; P = 5.22×10^−2^	0.06±0.02 t_5143_ = 3.96; P = 7.50×10^−5^
	q-values sum (*β*_∑Q_)	0.22±0.09 t_5077_ = 2.43; P = 1.52×10^−2^	0.06±0.02 t_5143_ = 2.65; P = 7.98×10^−3^

#### Assessing the consequences of the valence-induced confidence bias

**Fig 7:**
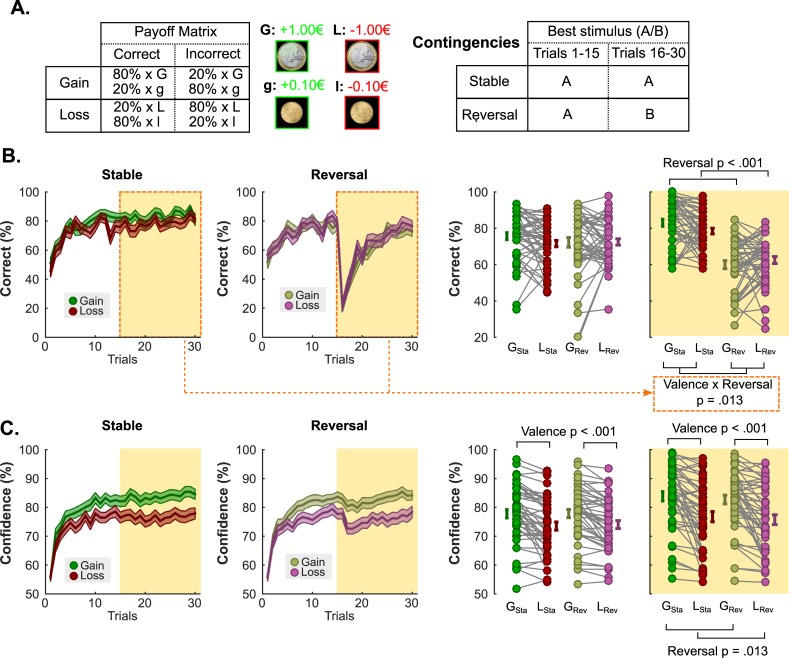
**Experiment 3 task schematic, reversal learning and confidence results. (A) Task design and contingencies.** (B) **Performance.** Trial by trial percentage of correct responses in the partial (left) and the complete (middle-left) information conditions. Filled colored areas represent mean ± sem; Middle-right and right: Individual averaged performances in the different conditions, before (middle-right) and after (right) the reversal. The orange shaded area highlights the post-reversal behavior. Connected dots represent individual data points in the within-subject design. The error bar displayed on the side of the scatter plots indicate the sample mean ± sem. (C) **Confidence.** Trial by trial confidence ratings in the partial (left) and the complete (middle-left) information conditions. Filled colored areas represent mean ± sem; Middle-right and right: Individual averaged performances in the different conditions, before (middle-right) and after (right) the reversal. The orange shaded area highlights the post-reversal behavior. Connected dots represent individual data points in the within-subject design. The error bar displayed on the side of the scatter plots indicate the sample mean ± sem. G_Sta_: Gain Stable; L_Sta_: Loss Stable; G_Rev_: gain reversal; L_Rev_: Loss Reversal.

We finally investigated potential consequences of the valence-induced confidence bias. We reasoned that, in volatile environments, confidence could be the meta-cognitive variable underlying decisions about whether to adjust learning strategies. In this case, individuals should exhibit lower performance in loss than gain contexts when contingencies are stable and better performance when contingencies change. The reason is that, because confidence is lower in loss contexts, they should sub-optimally explore alternative strategies when contingencies are stable but should display greater ease to change/adjust their learning strategies when contingencies change. To test this hypothesis, we invited 48 participants to partake in a reversal-learning task, where the probabilistic outcomes associated with half of the pairs saw their contingencies reversing halfway through the task (see **Methods**). Importantly, participants were explicitly told that the environment was unstable, so that strategies might need to be adjusted. Similar to experiments 1 and 2, outcomes could be either gains or losses depending on the pairs, and participants had to indicate how confident they felt about their choices (**Fig 7A**).

In the first half of the task (i.e. before the occurrence of any reversal), replicating our previous findings, we found that while learning performance was unaffected by the outcome valence (ANOVA; main effect of reversal F_1,47_ = 0.64; P = 0.42; main effect of valence F_1,47_ = 2.46; P = 0.12; interaction F_1,47_ = 2.38; P = 0.13; **Fig 7B**), confidence ratings were (ANOVA; main effect of reversal F_1,47_ = 0.66; P = 0.42; main effect of valence F_1,47_ = 39.13; P = 1.10×10^−7^; interaction F_1,47_ = 0.42; P = 0.52; **Fig 7C**). Yet and most importantly, in the second half of the task (i.e. after reversals happened in Reversal contexts), we observed an interaction between the Valence and Reversal factors on performance (ANOVA; main effect of reversal F_1,47_ = 88.67; P = 2.15×10^−12^ main effect of valence F_1,47_ = 0.26; P = 0.62; interaction F_1,47_ = 6.69; P = 0.01; **Fig 7A**). Post-hoc tests confirmed that participants performed relatively better in the gain than in the loss conditions if no reversal occurred (gain vs loss: t-test t_47_ = 2.34; P = 0.02), and showed a non-significant tendency to perform better in the loss than in the gain contexts if a reversal happened (gain vs loss: t-test t_47_ = -1.17; P = 0.25). Overall, these results indicate that the performance benefits for the gain frame in the stable context are eliminated in the reversal context, which seems to confirm our hypothesis that a valence-induced bias in confidence bears functional consequences.

## Discussion

In this paper we investigated the effect of context-value on confidence during reinforcement-learning, by combining well-validated tasks: a probabilistic instrumental task with monetary gains and losses as outcomes [8,32,35], and two variants of a confidence elicitation task [40,47]: a free elicitation of confidence (experiment 1), and an incentivized elicitation of confidence called matching probability (experiment 2). Behavioral results from two experiments consistently show a clear dissociation of the effect of decision frame on learning performance and confidence judgments: while the valence of decision outcomes (gains vs. losses) had no effect on the learning performance, it significantly impacted subjects’ confidence in the very same choices. Specifically, learning to avoid losses generated lower confidence reports than learning to seek gains regardless of the confidence elicitation methods employed. These results extend prior findings [31], by demonstrating a biasing effect of incentive valence in a reinforcement learning context. They are also consistent with other decision-making studies reporting that positive psychological factors and states, such as joy or desirability, bias confidence upwards, while negative ones, such as worry, bias confidence downwards [26,28–30].

Based on the current design and results, we can rule out two potential explanation for the presence of this confidence bias. First, we used both a free confidence elicitation method (experiment 1) and an incentivized method (experiment 2) and clearly replicate our results across these two methods. This indicates that the confidence bias cannot be attributed to the confidence elicitation mechanism. This is also supported by the fact that the confidence bias is observed despite the incentives in the primary task (gain and loss) being orthogonalized from the ones used to elicit confidence judgments (always framed as a gain). Second, an interesting feature of the present experiments is that monetary outcomes are displayed after–rather than before- confidence judgments. At the time of decision and confidence judgments, the value of decision-contexts is implicitly inferred by participants and not explicitly displayed on the screen. Combined with the fact that loss and gain conditions were interleaved and that previous studies indicate that in a similar paradigm subjects remain largely unaware of the contextual manipulations [48], this suggests that the biasing effect of monetary outcomes demonstrated in previous reports [31] is not due to a simple framing effect, created by the display of monetary gains or losses prior to confidence judgments.

Contrary to our previous study [31], the current reinforcement-learning design provides little control on the effect of the experimental manipulations on choice reaction times. Our results show that, like confidence, reaction times are also biased by the context value. Given that some studies have suggested that reaction times could inform confidence judgments [45]–although this has recently been challenged [49]-, the observed confidence bias could be a by-product of a reaction-time bias. However, both our control analysis (**Table 5**) and our previous study [31] seem to rule out this interpretation and point toward an authentic confidence bias that is at least partially independent of reaction times.

We offer two interpretations for the observed effects of gains versus losses on confidence. In the first interpretation, we propose that loss prospects simply bias confidence downward. In the second interpretation, we propose that loss prospects improve confidence calibration over gain prospects, thereby correcting overconfidence. Following the first interpretation, the apparent improvement in confidence calibration observed in our study does not correspond to a confidence judgment improvement *per se*, but is a mere consequence of participants being overconfident in this task. Accordingly, in a hypothetical task where participants would be underconfident in the gain domain, while the loss prospects would aggravate this underconfidence under the first interpretation, they would improve confidence calibration (hence correct this underconfidence) under the second interpretation. Future research is needed to distinguish between the two potential mechanisms.

Regardless of the interpretation of the reported effects, we showed that confidence can be modelled as a simple linear and additive combination of three variables: previous confidence rating, choice difficulty and the context value inferred from the context-dependent reinforcement learning model. The critical contribution of the present study is the demonstration that confidence judgments are affected by the value of the decision-context, also referred to as context value. The context value is a subjective estimate of the average expected-value of a pair of stimuli: in our experimental paradigm, the context value is therefore neutral (equal to 0) at the beginning of learning, and gradually becomes positive in the reward-seeking conditions and negative in the punishment-avoidance conditions [32]. The fact that the context-value significantly contributes to confidence judgments therefore complements our model-free results showing that outcome valence impacts confidence, while embedding it in the learning dynamics. The fact that the context value is a significant predictor of confidence judgments also suggests that context-dependency in reinforcement learning is not only critical to account for choice patterns but also to account for additional behavioral manifestations, such as confidence judgments and reaction times. This result therefore provides additional support for the idea that context values are explicitly represented during learning [32]. Crucially, context-dependency has been shown to display locally adaptive (i.e. successful punishment-avoidance in the learning test) and globally maladaptive (i.e. irrational preferences in the transfer test) effects [48]. Whether the context-dependence of confidence judgments is adaptive or maladaptive remains to be elucidated and will require teasing apart the different interpretation of this effect discussed above.

Our findings are also consistent with a growing literature showing that in value-based decision-making, choice-difficulty, as proxyed by the absolute difference in expected subjective value between the available [50–52] is a significant predictor of confidence judgments [42,43]. Finally, the notion that confidence judgments expressed in preceding trials could inform confidence expressed in subsequent trails is relatively recent, but has received both theoretical and experimental support [44,53] and intuitively echoes findings of serial dependence in perceptual decisions [54]. In interleaved experimental designs like ours, successive trials pertain to different learning contexts. Therefore, the significant serial dependence of confidence judgments revealed by our analyses captures a temporal stability of confidence, which is context-independent. This result is highly consistent with the findings reported in Rahnev and colleagues (2015), which show that serial dependence in confidence can even be observed between different tasks.

In the present report, the modelling approach is strongly informed and constrained by our previous studies [31,32]. In this sense, the proposed models are solely meant to provide a parsimonious, descriptive account of the confidence bias observed in the reinforcement-learning task. We acknowledge that other models and model families could provide a better, mechanistic and/or principled account of both learning performance and confidence judgments [1,23,55,56].

Overall, our results outline the importance of investigating confidence biases in reinforcement-learning. As outlined in the introduction, most sophisticated RL algorithms assume representation of uncertainty and/or strategy reliability estimates, which allow them to flexibly adjust learning strategies or to dynamically select among different learning strategies. Yet, despite their fundamental importance in learning, these uncertainty estimates have, so far, mostly emerged as latent variables, computed from individuals’ choices under strong computational assumptions [13,14,57–61]. In the present paper we propose that confidence judgments could be a useful experimental proxy for such estimates in RL. Confidence judgments indeed possess important properties, which suggest that they might be an important variable mitigating learning and decision-making strategies. First, confidence judgments accurately track the probability of being correct in stochastic environments, integrating expected and unexpected uncertainty in a close-to-optimal fashion [21,22]. Second, subjective confidence in one’s choices impacts subsequent decision processes [62] and information seeking strategies [63]. Finally, confidence acts as a common currency and therefore can be used to trade-off between different strategies [64,65].

With this in mind, biases of confidence could have critical consequences on reinforcement learning and reveal important features about the flexibility of learning and decision-making processes in different contexts. Along those lines, our last experiment provides suggestive evidence that, in volatile environments, the valence-induced confidence bias induces differences in learning-flexibility between reward-seeking and loss-avoidance contexts. The fact that such behavioral manifestations were absent in previous experiments—where participants were explicitly told that symbol-outcome association probabilities were stable—suggests that confidence is linked to a higher level of strategic exploration, contingent on the representation of task and environment structure. See also [21] for a similar claim in a sequence learning task.

Considering evolutionary perspectives, future research should investigate whether lower confidence in the loss domain–as demonstrated in the present report—could play an adaptive function, e.g. by allowing rapid behavioral adjustments under threat.

## Material and methods

### Ethics statement

All studies were approved by the local Ethics Committee of the Center for Research in Experimental Economics and political Decision-making (CREED), at the University of Amsterdam. All subjects gave informed consent prior to partaking in the study.

### Subjects

The subjects were recruited from the laboratory's participant database (www.creedexperiment.nl). A total of 84 subjects took part in this study: 18 took part in experiment 1 (8/10 M/F, age = 24.6±8.5), 18 in experiment 2 (8/10 MF, age = 24.6±4.3), and 48 in experiment 3 (26/22 M/F, age = 22.8±4). They were compensated with a combination of a base amount (5€), and additional gains and/or losses depending on their performance during the learning task: experiment 1 had an exchange rate of 1 (in-game euros = payout); experiments 2 and 3 had an exchange rate of 0.3 (in game euros = 0.3 payout euros). In addition, in experiments 2 and 3, three trials (one per session) were randomly selected for a potential 5 euros bonus each, attributed based on the confidence incentivization scheme (see below).

### Power analysis and sample size determination

Power analysis were performed with GPower.3.1.9.2 [66]. The sample size for Experiments 1 and 2 was determined prior to the start of the experiments based on the effects of incentives on confidence judgments in Lebreton et al. (2018). Cohen’s d was estimated from a GLM d = .941 t_23_ = 4.61, P = 1.23e-4). For a similar within-subject design, a sample of N = 17 subjects was required to reach a power of 95% with a two-tailed one-sample t-test.

### Learning tasks

#### Learning tasks–general features

All tasks were implemented using MatlabR2015a (MathWorks) and the COGENT toolbox (http://www.vislab.ucl.ac.uk/cogent.php). In all experiments, the main learning task was adapted from a probabilistic instrumental learning task used in a previous study [32]. Invited participants were first provided with written instructions, which were reformulated orally if necessary. They were explained that the aim of the task was to maximize their payoff and that gain seeking and loss avoidance were equally important. In each of the three learning sessions, participants repeatedly faced four pairs of cues—taken from Agathodaimon alphabet, corresponding to four conditions of a 2×2 factorial design. In all tasks, one of the factors was the Valence of the outcome, with two pairs corresponding to reward conditions, and two to loss conditions (the stimuli visuals were randomized across subjects). The second factor differed between Experiment 1–2 and Experiment 3, and is therefore detailed in the following sections.

#### Learning task conditions—Experiments 1 and 2

In experiments 1 and 2, the four cue pairs were presented 24 times in a pseudo-randomized and unpredictable manner to the subject (intermixed design). Within each pair the cues were associated with two possible outcomes defined by the valence factor (1€/0€ for the Gain and -1€/0€ for the Loss conditions in Exp. 1; 1€/0.1€ for the gain and -1€/-0.1€ for the loss conditions in Exp. 2) with reciprocal (but independent and fixed) probabilities (75%/25% and 25%/75%). The second factor was the Information given about the outcome: in Partial information trials, only the outcome linked to the chosen cue was revealed, while in Complete information trials, the outcome linked to both the chosen and unchosen cue were revealed.

Replacing the neutral outcome (0 euro) with a 10c gain or loss in Experiment 2 was meant to neutralize an experimental asymmetry between the gain and loss conditions, present in Experiment 1, which could have contributed to the valence impact on confidence in the partial information condition: when learning to avoid losses, subjects increasingly selected the symbol associated with a neutral outcome (0 euro), hence were provided more often with this ambiguous feedback. It is worth noting that this asymmetry was almost absent in the complete feedback case where the context value can be inferred in both gains and losses thanks to the counterfactual feedback (e.g. a forgone loss), and nonetheless showed lower reported confidence. Besides, despite this theoretical asymmetry in the partial condition, there was no detectable difference in performance between gain and loss performance in the partial information condition in the Experiment 1. Yet, replacing the ambiguous neutral option with small monetary gains and losses in experiment 2 completely corrected the imbalance between the partial information gain and loss conditions.

Participants were explicitly informed that there were fixed “good” and “bad” options within each pair, hence that the contingencies were stable.

#### Learning task conditions–experiment 3 (reversal)

In experiment 3, the four cue pairs were presented 30 times in a pseudo-randomized and unpredictable manner to the subject (intermixed design). Within each pair, the cues were associated with two possible outcomes defined by the Valence factor (1€/0.1€ for the Gain and -1€/-0.1€ for the Loss conditions) with reciprocal (but independent) probabilities (80%/20% and 80%/20%). The second factor was the presence or absence of a Reversal: in Reversal conditions, the probabilistic contingencies within a pair reversed halfway through the task (from the 16^th^ occurrence of the pair). Then, the initial “good” cue of a pair became the “bad” cue, and vice-versa. Instead, in Stable conditions, there was no such reversal, and the probabilistic contingencies remained stable throughout the task.

Note that participants were explicitly informed that “good” and “bad” options within each pair could change, hence that the contingencies were not always stable.

#### Learning task trials–all experiments

At each trial, participants first viewed a central fixation cross (500-1500ms). Then, the two cues of a pair were presented on each side of this central cross. Note that the side in which a given cue of a pair was presented (left or right of a central fixation cross) was pseudo-randomized, such as a given cue was presented an equal number of times on the left and the right of the screen. Subjects were required to select between the two cues by pressing the left or right arrow on the computer keyboard, within a 3000ms time window. After the choice window, a red pointer appeared below the selected cue for 500ms. Subsequently, participants were asked to indicate how confident they were in their choice. In Experiment 1, confidence ratings were simply given on a rating scale without any additional incentivization. In Experiments 2–3 confidence ratings were given on a probability rating scale and were incentivized (see below). To perform this rating, subjects could move a cursor–which appeared at a random position- to the left or to the right using the left and right arrows, and validate their final answer with the spacebar. This rating step was self-paced. Finally, an outcome screen displayed the outcome associated with the selected cue, accompanied with the outcome of the unselected cue if the pair was associated with a complete-feedback condition (only in Experiments 1–2).

#### Experiments 2 and 3—Matching probability and incentivization

In Experiment 2 and 3, participant’s reports of confidence were incentivized via a matching probability procedure that is based on the Becker-DeGroot-Marshak (BDM) auction [37] Specifically, participants were asked to report as their confidence judgment their estimated probability (p) of having selected the symbol with the higher average value, (i.e. the symbol offering a 75% chance of gain (G75) in the gain conditions, and the symbol offering a 25% chance of loss (L25) in the loss conditions) on a scale between 50% and 100%. A random mechanism, which draws a number (r) in the interval [0.5 1], is then implemented to select whether the subject will be paid an additional bonus of 5 euros as follows: If p ≥ r, the selection of the correct symbol will lead to a bonus payment; if p < r, a lottery will determine whether an additional bonus is won. This lottery offers a payout of 5 euros with probability r and 0 with probability 1-r. This procedure has been shown to incentivize participants to truthfully report their true confidence regardless of risk preferences [47,67].

Participants were trained on this lottery mechanism and informed that up to 15 euros could be won and added to their final payment via the MP mechanism applied on one randomly chosen trial at the end of each learning session (3×5 euros). Therefore, the MP mechanism screens (**Fig 3A**) were not displayed during the learning sessions.

#### Experiments 1–3—Transfer task

In all experiments, the 8 abstract stimuli (2×4 pairs) used in the third (i.e. last) session were re-used in the transfer task. All possible pair-wise combinations of these 8 stimuli (excluding pairs formed by two identical stimuli) were presented 4 times, leading to a total of 112 trials [7,32,34,68]. For each newly formed pair, participants had to indicate the option which they believed had the highest value, by selecting either the left or right option via button press in a manner equivalent to the learning task. Although this task was not incentivized, which was clearly explained to participants, they were nonetheless encouraged to respond as if money was at stake. In order to prevent explicit memorizing strategies, participants were not informed that they would have performed this task until the end of the third (last) session of the learning test.

### Model-free statistics

All model-free statistical analyses were performed using Matlab R2015a. All reported p-values correspond to two-sided tests. T-tests refer to a one sample t-test when comparing experimental data to a reference value (e.g. chance: 0.5), and paired t-tests when comparing experimental data from different conditions. ANOVA are repeated measure ANOVAs.

### Computational modelling—Experiments 1 and 2

#### Reinforcement-learning model

The approach for the reinforcement-learning modelling is identical to the one followed in Palminteri and colleagues (2015). Briefly, we adapted two models inspired from classical reinforcement learning algorithms [1]: the ABSOLUTE and the RELATIVE model. In the ABSOLUTE model, the values of available options are learned in a context-independent fashion. In the RELATIVE models, however, the values of available options are learned in a context-independent fashion.

In the ABSOLUTE model, at each trial t, the chosen (c) option value of the current context s is updated with the Rescorla-Wagner rule [3]:

$$Q_{t+1}(s,c)=Q_{t}(s,c)+\alpha_{c}\;\delta_{c,t}$$

$$Q_{t+1}(s,u)=Q_{t}(s,u)+\alpha_{u}\;\delta_{u,t}$$

Where *α*_*c*_ is the learning rate for the chosen (c) option and *α*_*u*_ t0he learning rate for the unchosen (u) option, i.e. the counterfactual learning rate. *δ*_*c*_ and *δ*_*u*_ are prediction error terms calculated as follows:

$$\delta_{c,t}=R_{c,t}-Q_{t}(s,c)$$

$$\delta_{u,t}=R_{u,t}-Q_{t}(s,u)$$

*δ*_*c*_ is updated in both partial and complete feedback contexts and *δ*_*u*_ is updated in the complete feedback context only.

In the RELATIVE model, a choice context value (*V*(*s*)) is also learned and used as the reference point to which an outcome should be compared before updating option values.

Context value is also learned via a delta rule:

$$V_{t+1}(s)=V_{t}(s)+\alpha_{V}\;\delta_{V,t}$$

Where *α*_*V*_ is the context value learning rate and *δ*_*V*_ is a prediction error-term calculated as follows: if a counterfactual outcome *R*_*U*,*t*_ is provided

$$\delta_{V,t}=(R_{c,t}+R_{U,t})/2-V_{t}(s),$$

If a counterfactual outcome *R*_*U*,*t*_ is not, provided, its value is replaced by its expected value *Q*_*t*_(*s*,*u*), hence

$$\delta_{V,t}=(R_{c,t}+Q_{t}(s,u))/2-V_{t}(s).$$

The learned context values are then used to center the prediction-errors, as follow:

$$\delta_{c,t}=R_{c,t}-V_{t}(s)-Q_{t}(s,c)$$

$$\delta_{u,t}=R_{u,t}-V_{t}(s)-Q_{t}(s,u)$$

In both models, the choice rule was implemented as a softmax function:

*P*_*t*_(*s*,*a*) = (1+exp(*β*(*Q*_*t*_(*s*,*b*)−*Q*_*t*_(*s*,*a*))))^−1^, where *β* is the inverse temperature parameter.

#### Model fitting

Model parameters *θ*_*M*_ were estimated by finding the values which minimized the negative log likelihood of the observed choice *D* given the considered model *M* and parameter values (−log(*P*(*D*|*M*,*θ*_*M*_))) and (in a separate optimization procedure) the negative log of posterior probability over the free parameters (−log(*P*(*θ*_*M*_|*D*,*M*))).

The negative logarithm of the posterior probability was computed as

$$-\log\left(P(\theta_{M}|D,M)\right)\propto -\log\left(P(D|M,\theta_{M})\right)-log(P(\theta_{M}|M))$$

where, *P*(*D*|*M*,*θ*_*M*_) is the likelihood of the data (i.e. the observed choice) given the considered model M and parameter values *θ*_*M*_, and *P*(*θ*_*M*_|*M*) is the prior probability of the parameters.

Following [32], the prior probability distributions *P*(*θ*_*M*_|*M*) assumed learning rates beta distributed (betapdf(parameter,1.1,1.1)) and softmax temperature gamma-distributed (gampdf(parameter,1.2,5)).

Note that the observed choices include both choices expressed during the learning test and choices observed during the transfer test, which were modelled using the option’s Q-values estimated at the end of learning. The parameter search was implemented using Matlab’s *fmincon* function, initialized at multiple starting points of the parameter space [69].

Negative log-likelihoods corresponding to the best fitting parameters (nLL_max_) were used to compute the Akaike’s information criterion (AIC) and the Bayesian information criterion (BIC). Similarly negative log of posterior probabilities corresponding to the best fitting parameters (nLPP_max_) were used to compute the Laplace approximation to the model evidence (ME) [69].

#### Model comparison

We computed at the individual level (random effects) the Akaike’s information criterion (AIC),

$$AIC=2df+2\times {nLL}_{max};$$

the Bayesian information criterion (BIC),

$$BIC=2\;\log\left(ntrials\right)\times df+2\times {nLL}_{max}$$

and the Laplace approximation to the model evidence (ME);

$$ME=-{nLPP}_{max}+\frac{df}{2}\log\left(2\pi \right)-\frac{1}{2}log|H|$$

Where df is the number of model parameters, and |*H*| is the determinant of the Hessian.

Individual model comparison criteria (AIC, BIC, ME) were fed to the mbb-vb-toolbox (https://code.google.com/p/mbb-vb-toolbox/) [70]. This procedure estimates the expected frequencies of the model (denoted EF) and the exceedance probability (denoted XP) for each model within a set of models, given the data gathered from all subjects. Expected frequency quantifies the posterior probability, i.e., the probability that the model generated the data for any randomly selected subject. Note that the three different criteria (AIC, BIC, ME) led to the same model comparison results.

#### Confidence model

To model confidence ratings, we used the parameter and latent variables estimated from the best fitting Model (i.e. the RELATIVE model) under the LPP maximization procedure. Note that for Experiment 1, confidence ratings were linearly transformed from 1:10 to 50:100%.

Following the approach taken with the RL models, we designed two models of confidence: the FULL and the REDUCED confidence models.

In the FULL confidence model, confidence ratings at each trial *t* (*c*_*t*_) were modelled as a linear combination of the choice difficulty–proxied by the absolute difference between the two options expected value (*dQ*_*t*_), the learned context value (*V*_*t*_), and the confidence expressed at the preceding trial (*c*_*t*−1_).

$$c_{t}=\beta_{0}+\beta_{dQ}\times {\Delta Q}_{t}+\beta_{V}\times V_{t}+\beta_{c1}\times c_{t-1},$$

where

$${\Delta Q}_{t}={abs(Q}_{t}(s,b)-Q_{t}(s,a))$$

and *β*_0_, *β*_*dQ*_, *β*_*V*_ and *β*_*c*1_ represents the linear coefficients of regression to be estimated.

In the REDUCED confidence model 1, we omitted the learned context value (*V*_*t*_), leading to

$$c_{t}=\beta_{0}+\beta_{\Delta Q}\times {\Delta Q}_{t}+\beta_{c1}\times c_{t-1},$$

In the REDUCED confidence model 2, we omitted the confidence expressed at the preceding trial (*c*_*t*−1_), leading to

$$c_{t}=\beta_{0}+\beta_{\Delta Q}\times {\Delta Q}_{t}+\beta_{V}\times V_{t}$$

Those models were implemented as generalized linear mixed-effect (glme) models, including subject level random effects (intercepts and slopes for all predictor variables). The models were estimated using Matlab’s *fitglme* function, which maximize the maximum likelihood of observed data under the model, using the Laplace approximation.

Modelled confidence ratings (i.e. confidence model fits) were estimated using Matlab’s *predict* function.

Because the REDUCED models are nested in the FULL model, a likelihood ratio test can be performed to assess whether the FULL model gives a better account of the data, while being penalized for its additional degrees-of-freedom (i.e. higher complexity). This test was performed using Matlab’s *compare* function.

To assess the specificity of V(s) we run two additional glmes including ∑*Q*_*t*_ = *Q*_*t*_(*s*,*b*) + *Q*_*t*_(*s*,*a*) and the reaction time, respectively as model-based and model-free variables affected by the valence factor. We tested whether in these glmes V(s) still predicted confidence rating despite sharing common variance with these variables.

Note that confidence is often explicitly modelled as the probability of being correct [17,18,23]. In our dataset, replacing Δ*Q*_*t*_ with the probability of choosing the correct option (*P*_*t*_(*s*,*correct*)) in the FULL confidence model gave very similar results on all accounts. Bayesian model comparisons indicate that these two models (i.e. including Δ*Q*_*t*_ or *P*_*t*_(*s*,*correct*) as independent variables) are not fully discriminable, but that the FULL model using Δ*Q*_*t*_ appears to give a slightly better fit of confidence ratings (exceedance probability in favor of GLM1, experiment 1: 77.95%; experiment 2: 69.79%).
